# Growth Arrest-Specific Factor 6 (GAS6) Is Increased in COVID-19 Patients and Predicts Clinical Outcome

**DOI:** 10.3390/biomedicines9040335

**Published:** 2021-03-26

**Authors:** Albert Morales, Silvia Rojo Rello, Helena Cristóbal, Aida Fiz-López, Elisa Arribas, Montserrat Marí, Anna Tutusaus, Paloma de la Cal-Sabater, Gerry A.F. Nicolaes, José T. Ortiz-Pérez, David Bernardo, Pablo García de Frutos

**Affiliations:** 1 Department of Cell Death and Proliferation, IIBB-CSIC, IDIBAPS, 08036 Barcelona, Spain; amorales@clinic.cat (A.M.); helena.cristobal@iibb.csic.es (H.C.); monmari@clinic.cat (M.M.); anna.tutusaus@iibb.csic.es (A.T.); 2 Barcelona Clinic Liver Cancer (BCLC) Group, Liver Unit, Hospital Clínic, CIBEREHD, 08036 Barcelona, Spain; 3 Servicio de Microbiología, Hospital Clínico Universitario de Valladolid, 47003 Valladolid, Spain; 12393921A@saludcastillayleon.es; 4 Mucosal Immunology Lab, Unidad de Excelencia Instituto de Biomedicina y Genética Molecular (IBGM), Universidad de Valladolid—CSIC, 47003 Valladolid, Spain; aidafizlopez@gmail.com (A.F.-L.); elisarribas@usal.es (E.A.); calsabater89@gmail.com (P.d.l.C.-S.); David.bernardo@uva.es (D.B.); 5 Department of Biochemistry, Cardiovascular Research Institute Maastricht (CARIM), Maastricht University, 6200 MD Maastricht, The Netherlands; g.nicolaes@maastrichtuniversity.nl; 6 Clinic Cardiovascular Institute, Hospital Clinic Barcelona, 08036 Barcelona, Spain; jtortiz@clinic.cat; 7 Centro de Investigaciones Biomédicas en Red de Enfermedades Hepáticas y Digestivas (CIBERehd), 28029 Madrid, Spain; 8 Centro de Investigación Biomédica en Red Sobre Enfermedades Cardiovasculares (CIBERCV), 28029 Madrid, Spain; 9 Department of Cell Death and Differentiation, Institut d’Investigacions Biomèdiques de Barcelona, IIBB-CSIC, Rosselló 161, 6th Floor, 08036 Barcelona, Spain

**Keywords:** COVID-19, AXL, MERTK, GAS6, viral infection, immune response, immunothrombosis, vitamin K

## Abstract

Background: Growth arrest-specific factor 6 (GAS6) and the Tyro3, AXL, and MERTK (TAM) receptors counterbalance pro-inflammatory responses. AXL is a candidate receptor for SARS-CoV-2, particularly in the respiratory system, and the GAS6/AXL axis is targeted in current clinical trials against COVID-19. However, GAS6 and TAMs have not been evaluated in COVID-19 patients at emergency admission. Methods: Plasma GAS6, AXL, and MERTK were analyzed in 132 patients consecutively admitted to the emergency ward during the first peak of COVID-19. Results: GAS6 levels were higher in the SARS-CoV-2-positive patients, increasing progressively with the severity of the disease. Patients with initial GAS6 at the highest quartile had the worst outcome, with a 3-month survival of 65%, compared to a 90% survival for the rest. Soluble AXL exhibited higher plasma concentration in deceased patients, without significant differences in MERTK among SARS-CoV-2-positive groups. GAS6 mRNA was mainly expressed in alveolar cells and AXL in airway macrophages. Remarkably, THP-1 human macrophage differentiation neatly induces AXL, and its inhibition (bemcentinib) reduced cytokine production in human macrophages after LPS challenge. Conclusions: Plasma GAS6 and AXL levels reflect COVID-19 severity and could be early markers of disease prognosis, supporting a relevant role of the GAS6/AXL system in the immune response in COVID-19.

## 1. Introduction

COVID-19 is characterized by the induction of an uncontrolled release of cytokines [1]. This is an important aspect of the disease as systemic immune overactivation due to SARS-CoV-2 infection causes the cytokine storm which can become life-threatening for the patient [2,3]. The body possesses restorative mechanisms, early triggered by pathogen and damage-associated molecular patterns (PAMPs and DAMPs), to counterbalance this pro-inflammatory response. One such protection system consists of vitamin K-dependent plasma proteins, which require carboxylation to become biologically active, and that serve as ligands for tyrosine kinase receptors of the Tyro3, AXL, and MERTK (TAM) subfamily [4,5]. The system consists of two soluble ligands, protein S (PROS1) and growth arrest-specific factor 6 (GAS6), and three membrane protein kinase receptors, (Tyro3, AXL, and MERTK). Components of the GAS6/TAM system increase in a diverse spectrum of inflammatory conditions [6], including septicemia and septic shock; but also in systemic inflammatory response syndrome (SIRS) in the absence of infection [7]. 

In several studies, plasma GAS6 concentration upon admission to intensive care is correlated with the severity of organ damage and acute respiratory distress syndrome [8,9,10,11]. Furthermore, recent data describe an interaction of the AXL receptor with the N-terminal domain of the SARS-CoV-2 spike glycoprotein [12]. This is particularly important, as ACE2 receptor expression is extremely low in the lower respiratory system, where AXL could have a prominent role. AXL inhibition is in clinical trial for COVID-19 treatment (ACCORD-2-001). Despite these facts, to date, no data have been reported on plasma concentrations of GAS6 and TAM receptors at the time of hospital admission of patients with COVID-19.

## 2. Materials and Methods

### 2.1. Patients

We prospectively measured the concentration of GAS6, sAXL and sMERTK in 132 consecutive patients admitted to the emergency ward of “Hospital Clínico Universitario de Valladolid” (Valladolid, Spain). The study was approved by the CEIm of the “Area de Salud Valladolid Este” PI20-1716, with the required signed informed consent. COVID-19 diagnosis was established by PCR for SARS-CoV-2 in airway secretion performed at admission. Individual demographic and clinical data from each group are included as Supplemental Data.

### 2.2. Cell Culture and Treatments

THP-1 human monocytic cells (Sigma-Aldrich, St. Louis, MO, USA) were activated to macrophages with phorbol 12-myristate 13-acetate (PMA). In brief, THP-1 cells were counted and seeded in 12-well plates (250.000 cells/well) in the presence of 100 ng/mL PMA for 4 days (in RPMI + 10% FBS). Afterwards, the PMA-containing media were removed; cells were washed and left overnight with RPMI + 2.5% FBS + 50 μM beta-mercaptoethanol. Then, THP-1 macrophages were pre-incubated with bemcentinib (1 µg/mL, BerGenBio AS, Bergen, Norway) for 60 min before adding LPS (50 ng/mL; E. coli 0111:B4 Sigma-Aldrich) for inflammatory activation for 2 hrs. Additionally, THP-1 cells were incubated with recombinant human GAS6 (500 ng/mL; 885-GS R&D Systems, Minneapolis, MN, USA) or activating goat antihuman AXL polyclonal antibody (10 nM; R&D AF154). Afterwards, total RNA was isolated with TRIzol reagent, reverse transcribed to complementary DNA (cDNA) using the iScript cDNA Synthesis Kit (BioRad, Hercules, CA, USA), following the manufacturer’s instructions. The housekeeping gene 18S was used as reference gene for normalization. To analyze THP-1 inflammatory features, the mRNA expression of IL-1beta, MCP1, TNF, and IL-6 were determined. 

The primers sequences used were:

MCP1 (NM_002982): 

5′-CCCCAGTCACCTGCTGTTAT-3′, 5′-TGGAATCCTGAACCCACTTC-3′

IL-1beta (NM_000576):

5′-TCAGCCAATCTTCATTGCTC-3′, 5′-GCCATCAGCTTCAAAGAACA-3′

TNF-alpha (NM_000594):

5′-TTTGATCCCTGACATCTGGA-3′, 5′-GGCCTAAGGTCCACTTGTGT-3′

18S (NM_022551):

5′-TCACTGAGGATGAGGTGGAA-3′, 5′-GCTTGTTGTCCAGACCATTG-3′

IL-6 (NM_00600):

5′-GACAGCCACTCACCTCTTCA-3′, 5′-CCTCTTTGCTGCTTTCACAC-3′

### 2.3. Quantitation of MERTK, AXL, and GAS6 in Plasma

Soluble MERTK (sMERTK), soluble AXL (sAXL), and GAS6 levels were determined in plasma from patients by the ELISA technique using commercial kits from R&D systems (DuoSet ELISA, McKinley, MN, USA) following the manufacturer’s instructions. Plasma samples were diluted 1:50 before determination (GAS6, sAXL) or 1:10 (sMERTK). These DuoSet ELISA kits, with the components required for the development of sandwich ELISAs, allow one to measure natural and recombinant human GAS6, sAXL and sMERTK proteins in cell culture supernatants, serum, plasma, or urine samples at physiopathological concentrations (kits reference and detection range: DY885B GAS6, 15.6–000 pg/mL; DY154 sAXL, 62.5–4000 pg/mL; DY6488 sMERTK, 78.1–5000 pg/mL).

### 2.4. Immunoblot Analysis

Cell lysates were prepared in RIPA buffer plus proteinase inhibitors. Samples containing 25 µg were separated by 10% SDS-PAGE. Proteins were transferred to nitrocellulose membranes, blocked in 5% nonfat milk for 1 h at room temperature, and incubated overnight at 4 °C with the primary antibodies: AXL (M20, sc-1097, Santa Cruz Biotechnologies, Dallas, TX, USA) 1:250 goat; β-actin-HRP (A3854, Sigma-Aldrich, St. Louis, MO, USA) 1:40,000 mouse.

### 2.5. mRNA Expressions of GAS6, AXL, and MERTK in Human Lung

Diagrams were graphed based on single-cell data from the Human Protein Atlas accessed on 18 January 2021 [13]: 

GAS6: www.proteinatlas.org/ENSG00000183087-GAS6/celltype/lung, accessed on 18 January 2021.

AXL: www.proteinatlas.org/ENSG00000167601-AXL/celltype/lung, accessed on 18 January 2021.

MERTK: www.proteinatlas.org/ENSG00000153208-MERTK/celltype/lung, accessed on 18 January 2021.

### 2.6. Statistical Analysis 

GraphPad Prism Software (version 4.02, La Jolla, CA, USA) was used to perform a one-way Anova analysis with Bonferroni’s multiple comparison as post-hoc test. Significance levels were defined as * *p* < 0.05; ** *p* < 0.01; and *** *p* < 0.001. In addition, survival data were graphed using Kaplan–Meier estimate and Logrank test for comparison among curves.

## 3. Results and Discussion

Increased circulating levels of GAS6 are detected after organ damage and several inflammatory settings. To evaluate its possible participation in COVID-19 we measured GAS6 in plasma samples from patients admitted consecutively to the emergency services of a single university hospital in Valladolid (Spain) during the first peak of the COVID-19 pandemic (from 27 to 31 March 2020). The population consisted entirely of local residents with a median age of 65 years (IQR; 54–77) and 51% female (Supplementary Table S1). Fifty-two patients tested negative for SARS-CoV-2 (NEG), while 80 were positive for the virus. The diagnosis was established by SARS-CoV-2 PCR in airway secretion. Individual data for each group are included in Supplemental Data.

The concentration of GAS6 in plasma was significantly higher in the SARS-CoV-2-positive group (19.3 ± 1.0 ng/mL vs. 11.6 ± 1.0 ng/mL; *p* < 0.001) than in negative patients. 

**Figure 1:**
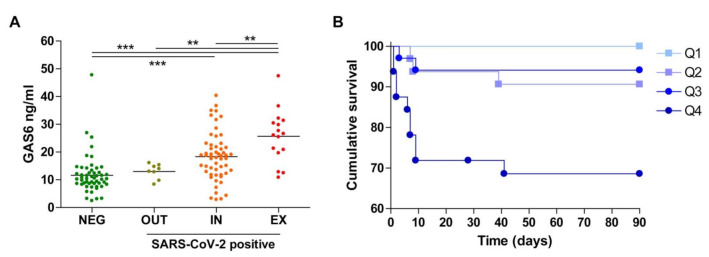
Plasma growth arrest-specific factor 6 (GAS6) concentration in consecutive emergency patients and survival curves related to quartile GAS6 values. (**A**) GAS6 plasma concentration was measured in patients (NEG, negative test for SARS-CoV-2; OUT, positive test not requiring hospital admission; IN, positive test and admission survivors; EX, positive test, deceased) and compared using one-way Anova. **, *p* < 0.01; ***, *p* < 0.001. (**B**) Survival plot of all patients divided by GAS6 concentration quartile at admission. Lowest quartile (Q1), second quartile (Q2), third quartile (Q3), and highest quartile (Q4). The GAS6 survival curves were different in a logrank test (*p* < 0.0001) and the Q4 survival was significantly different to all other curves (*p* = 0.004 vs. Q3; *p* = 0.017 vs. Q2 and *p* < 0.001 vs. Q1).

Next, we stratified SARS-CoV-2-positive patients based on their different outcomes. A group of patients did not require admission at the hospital (OUT; *n* = 8), while the rest required hospitalization (*n* = 72). Of the latter group, 56 patients survived up to 3 months of observation from hospital admission (IN), while 16 patients died (EX). When the values of GAS6 at admission were compared (Figure 1A), those patients who did not require hospitalization showed the lowest values (OUT, 13.0 ± 1.0 ng/mL), similar to the levels observed in SARS-CoV-2-negative patients. 

However, individuals that needed hospitalization and survived (IN; 18.3 ± 1.2 ng/mL; *p* = 0.043) and those who died (EX, 25.7 ± 2.4 ng/mL; *p* = 0.004) exhibited significantly higher plasma GAS6 concentrations than those of SARS-CoV-2-negative patients. Therefore, the amount of GAS6 in the plasma increased progressively with the severity of the disease in SARS-CoV-2-positive patients, increased GAS6 levels also being associated with the need for clinical admission. We then tested if the concentration of GAS6 at the initial admission could be predictive of the final outcome of the illness. We divided the group in quartiles according to their GAS6 values and compared the survival curves using a Cox regression analysis. The survival pattern is reflected in the Kaplan–Meier plot shown in Figure 1B. 

Those patients with the highest quartile of GAS6 concentration at the moment of admission had the worst outcome with a survival of 65% at 3 months, significantly different from the other groups that showed a survival of over 90%. Of note, in the lowest GAS6 quartile, all SARS-CoV-2-positive patients survived. 

**Figure 2:**
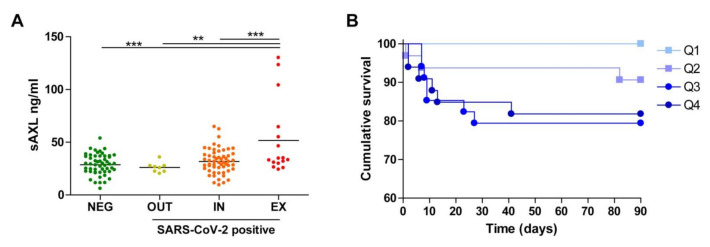
Plasma soluble AXL (sAXL) concentration in consecutive emergency patients and survival curves related to quartile AXL values. (**A**) sAXL plasma concentration was measured in all patients and compared using one-way Anova. **, *p* < 0.01; ***, *p* < 0.001. (**B**) Survival plot of all patients divided by sAXL quartile at admission. The sAXL survival curves were different in a logrank test (*p* < 0.048) and the Q4 survival was significantly different only to the Q1 curve (*p* = 0.011).

We also measured sAXL and sMERTK, the products of the proteolytic shedding of the main receptors for GAS6 found in plasma. For sAXL, only the group of SARS-CoV-2-positive patients that did not survive during the initial 90 days (EX) had a significantly higher concentration in plasma compared to the SARS-CoV-2-negative and surviving positive groups (Figure 2A). Nevertheless, patients admitted to the emergency ward with higher sAXL values in plasma showed worse survival than those with lower concentration, although this association was not as strong as that observed for the GAS6 highest quartile (Figure 2B). 

**Figure 3:**
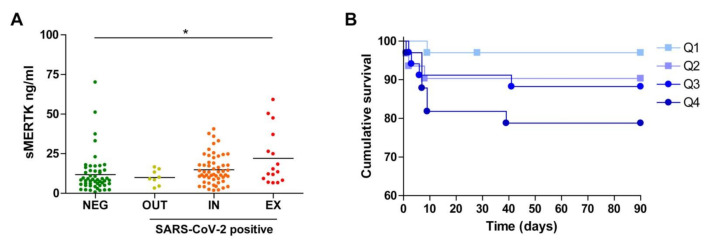
Plasma soluble MERTK(sMERTK) concentration in consecutive emergency patients and survival curves related to quartile MERTK values. (**A**) sMERTK plasma concentration was measured in all patients and compared using one-way Anova. *, *p* < 0.05. (**B**) Survival plot of all patients divided by sMERTK concentration quartile at admission. The MERTK survival curves were not different in a logrank test (*p* = 0.154) and the Q4 survival was significantly different only to the Q1 curve (*p* = 0.024 vs. Q1).

In contrast, no significant differences were observed in sMERTK concentration among the COVID-19-positive groups (Figure 3A), although the group of SARS-CoV-2-positive patients that died during the initial 90 days (EX) displayed higher sMERTK levels than the SARS-CoV-2-negative group. In addition, a trend for worst outcome was also observed in the patients with higher sMERTK levels (Figure 3B). 

Considering that the samples were obtained at admission to the emergency ward, our results suggest that GAS6 is expressed as an early mechanism of response to the viral infection by SARS-CoV-2 and points to AXL as a possible receptor involved in COVID-19-related GAS6 signaling. 

**Figure 4:**
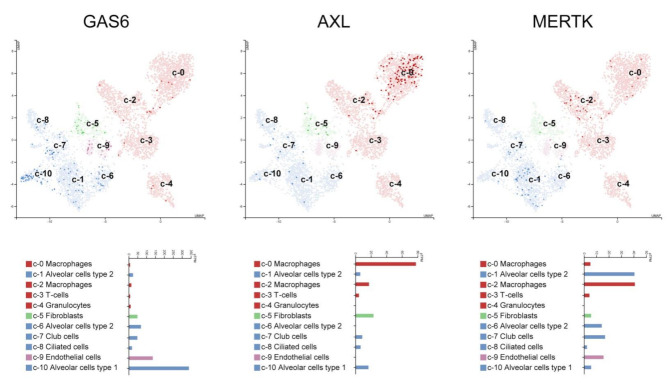
GAS6, AXL, and MERTK mRNA expression in human lung cells. Individual cells in each cluster are visualized with color intensity according to the specific mRNA expression; where each dot corresponds to a cell. Mean mRNA expression on each individual cell type is indicated below. Data are derived from http://www.proteinatlas.org.

To confirm the specific expression of these molecules on different cell types of the respiratory tract that may have a relevant role in COVID-19 development, we analyzed GAS6, AXL, and MERTK mRNA expression in a public database for single-cell RNAseq experiments [13]. As shown in Figure 4, GAS6 is mainly expressed in alveolar cells (type I and type II) and endothelial cells. 

Interestingly, AXL mRNA is noticeably more expressed in the macrophage cluster including airway/alveolar macrophages, in line with data on record [14], and to a lesser extent in fibroblasts. The high AXL expression in airway/alveolar macrophages suggests that this cell type is where AXL may play a more relevant role. 

**Figure 5:**
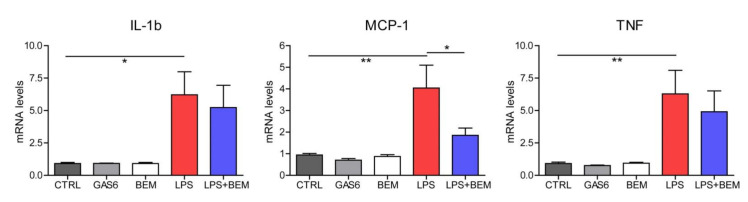
AXL inhibition reduced cytokine production upon LPS challenge in activated human macrophages (THP-1 cells). Phorbol 12-myristate 13-acetate (PMA)-activated THP-1 cells were exposed to LPS (50 ng/mL) and/or pre-incubated for 1 h with bemcentinib (1 µg/mL). mRNA levels of IL-1β, MCP-1, and TNF were determined 2 h later. A control group was stimulated with recombinant human 500 ng/mL GAS6. Groups were compared using one-way Anova. *, *p* < 0.05; **, *p* < 0.01.

To verify this point, we treated PMA-activated human THP-1 cells with LPS and quantified cytokine production. As expected, activated THP-1 cells exposed to LPS exhibited an induction of inflammatory genes such as IL-1b, TNF or MCP-1 (Figure 5).

Specific AXL inhibition with bemcentinib was enough to diminish cytokine expression upon LPS exposure, particularly decreasing MCP-1 levels significantly. Interestingly, MCP-1 has also been proposed as a biomarker associated with disease severity of COVID-19 [15], suggesting that MCP-1 reduction obtained after AXL inhibition may be involved in less recruitment of monocytes and thrombosis protection. 

Of note, GAS6 alone did not increase cytokine production, indicating that blockade of the GAS6/AXL axis is only required when the pro-inflammatory process has been triggered.

**Figure 6:**
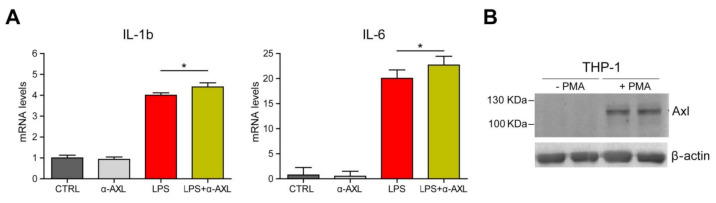
AXL overexpression in activated human macrophages (THP-1 cells) and effect of AXL-activating antibodies on LPS-induced cytokine production. (**A**) PMA-activated THP-1 cells were exposed to LPS (50 ng/mL) and/or pre-incubated for 1 h with an activating goat antihuman AXL polyclonal antibody (10 nM; R&D AF154). mRNA levels of IL-1b and IL-6 were determined 6 h later. Groups were compared using one-way Anova. *, *p* < 0.05. (**B**) AXL and β-actin protein expression in resting and PMA-activated THP-1 cells was analyzed.

Moreover, incubation of activated THP-1 cells with an anti-AXL activating antibody was only modestly effective to further increase cytokine induction by LPS (Figure 6A). Although IL-1b or IL-6 upregulation was detected after 6 h of LPS challenge, this was not significantly observed for MCP-1 or TNF. In this sense, the strong expression of AXL after PMA activation exhibited by THP-1 cells (Figure 6B) may justify the discreet effect of additional AXL activation by addition of GAS6 or AXL-activating antibodies. 

High AXL levels could be by themselves responsible for AXL autophosphorylation, as previously reported [16], or activated by endogenous GAS6 produced by THP-1 cells, making GAS6 exogenous addition less relevant in this context. Further experiments, including those with macrophages deficient in GAS6 and TAM receptors, will be necessary to corroborate this point, being out of the scope of our communication, which is principally devoted to pinpointing for the first time the relevant alteration of the GAS6/TAM system observed in COVID-19 patients. 

In other viral respiratory infections, GAS6 induction appears early after infection in animal models and its plasma concentration is maintained above the pre-infection concentration for weeks [17]. In this setting, as well as in many other models of inflammation/infection studied, GAS6/AXL represents a mechanism of control of the immune response in order to protect against organ damage resulting from the cytokine storm induced by intense inflammatory conditions. 

Ni et al. have shown that recombinant GAS6 infusion improves the outcome of septicemia in mice, controlling multi-organ dysfunction [18]. One of the target cells of GAS6 in this context is the vascular endothelium that exhibits reduced LPS-induced permeability in the presence of increased GAS6 concentrations. Interestingly, GAS6 is also necessary to maintain the response of vascular endothelium during inflammatory conditions, allowing endothelial cell interactions with platelets and leukocytes [19]. 

Moreover, as a consequence of the increase in GAS6 activity after viral infections downregulating the local inflammatory response, the host becomes susceptible to secondary bacterial infections [17]. Therefore, the increases in GAS6 and soluble TAM receptors not only may be indicative of COVID-19-associated risk but also a signal pointing to the GAS6/TAM system as a potential target for COVID-19 treatment. Interestingly, among the underlying medical conditions linked to severe COVID-19, there are several pathologies that frequently display increased GAS6/TAMs levels, such as liver cirrhosis [20], non-alcoholic steatohepatitis [21], and chronic kidney [22], pulmonary [23], or heart diseases [24]. Whether GAS6 or other TAM changes may be particularly detrimental in these specific COVID-19 patients is another aspect that merits pursuing. 

The increase observed in GAS6 underlines the importance of the vitamin K status in COVID-19. Interestingly, increased uncarboxylated matrix Gla protein has been detected in COVID-19 patients with poor outcomes [25], and regular use of oral antivitamin K anticoagulants prior to COVID-19 is associated with lower survival in elderly patients [26]. A poor vitamin K status could decrease the vitamin K-dependent actions of GAS6 and the anticoagulant PROS1, among other effects [27].

Of note, a recent publication has just observed a direct interaction of AXL with the N-terminal domain of SARS-CoV-2 spike glycoprotein [12], further increasing interest in the effect that the GAS6/AXL pathway may have on COVID-19 pathology. Interestingly, bemcentinib, which selectively inhibits AXL kinase, blocks viral entry and enhances the antiviral type I IFN response [27]. Bemcentinib, which has been reported effective in preclinical models against several enveloped viruses, including Ebola [28] and Zika virus [29], is now being evaluated in two Phase 2 studies for the treatment of COVID-19 in hospitalized patients (the UK national platform study-ACCORD2-EudraCT 2020-001736-95, UK; and the BerGenBio ASA study BGBC020 (CTRI-2020-10-028602), in India and South Africa). 

Supporting an antiviral effect, a recent oral presentation from Dr. Maury Lab at the Conference on Retroviruses and Opportunistic Infections (CROI, 6–10 March 2021) has revealed that AXL facilitates ACE-2-mediated endocytosis of SARS-COV-2, which was prevented by bemcentinib in vitro [30]. In addition, in a murine hepatitis betacoronavirus model, AXL inhibition reduced the viral load and significantly enhanced signatures of type I interferon response. These observations further justify the biomedical importance of GAS6/AXL intervention on COVID-19 treatment and highlight the relevance of GAS6/TAM serum data in COVID-19 patients for their potential role in clinical management.

**Figure 7:**
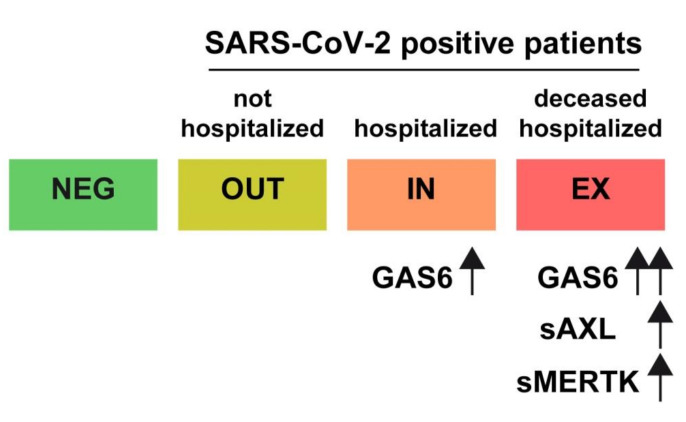
Schematic representation of early GAS6/AXL/MERTK changes in COVID-19 patients. Negative SARS-COV-2 patients and positive that did not require hospitalization exhibited no significant changes in GAS6-related proteins. Significant increase in GAS6 was observed in hospitalized patients, which were greater in deceased. Hospitalized patients that finally died also exhibited higher plasma concentration of sAXL and sMERTK.

In this sense, our results endorse the hypothesis that the concentration of GAS6 in the blood is proportional to the severity of the disease and could be an early marker of disease prognosis in COVID-19. In addition, levels of sAXL and sMERTK are significantly increased in deceased SARS-CoV-2-positive patients, suggesting that their measurement may provide evidence of a bad prognosis (Figure 7).

Moreover, we observed that the expression of the GAS6/AXL pathway in the lung suggests that GAS6 may be secreted and play a protective role in alveolar and endothelial cells, while AXL is principally expressed in macrophages. In fact, reduction of AXL levels diminishes the production of inflammatory cytokines by macrophages upon LPS challenge. Therefore, we conclude that SARS-CoV-2 induces an immune response in which the GAS6–TAM system of ligands and receptors is implicated. Further studies are required to identify specific individuals, mainly with previous chronic pathologies, that could be particularly at risk.

## 4. Conclusions

GAS6 serum levels in patients admitted to the emergency ward were higher in SARS-CoV-2-positive patients, increasing progressively with the severity of the disease. In addition, deceased COVID-19 patients also exhibited higher plasma concentration of sAXL and sMERTK at the time of emergency entry. Moreover, AXL inhibition reduced cytokine production upon LPS challenge in THP-1 human macrophages, which exhibited AXL overexpression upon activation. In summary, these results support a relevant role of the GAS6/AXL system in the immune response against COVID-19, suggesting them as early markers of disease prognosis and GAS6/AXL targeting as plausible clinical therapy for COVID-19 patients.

## Data Availability

Individual de-identified patient data are included in the document Supplemental data 1.

## Supplementary Materials

The following are available online at https://www.mdpi.com/article/10.3390/biomedicines9040335/s1. Supplementary Table S1: Demographic and baseline characteristics of 132 patients on admission to the Emergency Ward. Supplementary Data S1: De-identified patient data including GAS6, sAXL, and sMERTK individual values.
